# Molecular characterisation of equid alphaherpesvirus 1 strains isolated from aborted fetuses in Poland

**DOI:** 10.1186/s12985-018-1093-5

**Published:** 2018-12-03

**Authors:** Anna Karolina Matczuk, Małgorzata Skarbek, Natalia Anna Jackulak, Barbara Anna Bażanów

**Affiliations:** Division of Microbiology, Department of Pathology, Faculty of Veterinary Medicine, Wrocław University of Environmental and Life Sciences, Norwida 31, Wroclaw, 50-375 Poland

**Keywords:** EHV-1, Abortion in mares, Phylogenetic analysis, ORF30, ORF68, Equine herpesvirus myeloencephalopathy

## Abstract

**Background:**

Equid alphaherpesvirus 1 (EHV-1) is one of the main infectious causative agents of abortion in mares and can also be associated with stillbirth, neonatal foal death, rhinopneumonitis in young horses and a neurological disorder called equine herpesvirus myeloencephalopathy (EHM). The neuropathogenicity of the virus was shown to be significantly higher in EHV-1 strains that carry a single nucleotide point (SNP) mutation in the ORF30, which encodes a catalytic subunit of viral DNA polymerase (ORF30 D_752_). Another gene, ORF68 is frequently used for phylogenetic analysis of EHV-1.

**Methods:**

27 EHV-1 strains isolated from aborted equine fetuses in Poland, collected between 1993 and 2017, were subjected to PCR targeting the open reading frames (ORFs) 30 and 68 of the EHV-1 genome. PCR products obtained were sequenced and SNPs were analyzed and compared to sequences available in GenBank.

**Results:**

None of the analyzed sequences belonged to the ORF30 D_752_neuropathogenic genotype: all EHV-1 belonged to the non-neuropathogenic variant N_752_. On the basis of ORF68 sequences, the majority of EHV-1 sequences (76.9%) cannot be assigned to any of the known groups; only six sequences (23.1%) clustered within groups II and IV.

**Conclusions:**

EHV-1 strains obtained from abortion cases belong to the non-neuropathogenic genotype. Many EHV-1 ORF68 sequences have similar SNPs to those already described in Poland, but a clear geographical distribution was not observed. A single particular ORF68 sequence type was observed in strains isolated from 2001 onwards.

**Electronic supplementary material:**

The online version of this article (10.1186/s12985-018-1093-5) contains supplementary material, which is available to authorized users.

## Background

Equid alphaherpesvirus 1 (formerly called equine herpesvirus 1, EHV-1) is one of the main infectious causative agents of abortion in mares [1]. Infection with EHV-1 can also be associated with stillbirth, neonatal foal death, rhinopneumonitis in young horses and a neurological disorder called equine herpesvirus myeloencephalopathy (EHM) [2]. EHV-1 can cause primary infection, but reinfection with a new strain or reactivation from latency was also shown to induce disease [3].

Infections with EHV-1 remain a major problem in horse breading studs in Poland as well as in other European countries. Admittedly, vaccines are available, but do not provide complete protection against abortion or EHM in mares [4, 5]. Moreover, in recent years, an increase in the number of EHM cases has been observed in many countries, e.g., France, Germany, Ethiopia, Argentina and the USA [6–10].

EHV-1 belongs to the subfamily *Alphaherpesvirinae,* a family of *Herpesviridae*. Its linear, double-stranded DNA genome of about 150kbp contains 80 open reading frames (ORFs), four of which are duplicated [11]. Although the mechanism of EHM development is not well understood, the potential to cause neuropathogenicity is significantly higher in EHV-1 strains that carry a single nucleotide point (SNP) mutation in the ORF30, which encodes a catalytic subunit of viral DNA polymerase [12]. The A to G mutation in nucleotide (nt) position 2254 of the virus genome causes substitution of asparagine (N) by aspartic acid (D) at amino acid position 752 in the catalytic subunit of the viral DNA polymerase. This single amino acid mutation in the viral polymerase of EHV-1 causes higher tropism to lymphocytes and longer viremia in experimentally infected horses when compared to animals infected with EHV-1 lacking this particular mutation [13].

Although infection with N_752_ can also cause EHM, infections with D_752_ increase the risk of developing EHM [3, 6]. For this reason, EHV-1 N_752_ is referred to as a non-neuropathogenic genotype, and D_752_ as a neuropathogenic genotype. Other risk factors for EHM, beside the genotype of the virus, include host and environmental factors such as breed, age and sex of the horse [14].

In some neuropathogenic EHV-1 strains, another mutation, a substitution C to A in position 2258 in addition to A to G in position 2254 in the ORF30 gene was observed [15]. Infection with the neuropathogenic genotype of EHV-1 can also lead to severe abortion outbreaks [5]. Analysis of the whole EHV-1 genome sequences of reference neuropathogenic strain Ab4 and the less virulent V592 strain revealed that the highest variation rate occurs in the ORF68 gene, a homologue to the human herpes simplex virus type US2 region [12]. Analysis of 131 field isolates revealed that, indeed, this region of the EHV-1 genome has the highest mutation rate (2%), which allows its nucleotide sequence to be used as a genetic marker to classify virus strains into different groups [12]. The strains were divided into six groups (two strains remained unassigned) on the basis of SNPs in the polymorphic region of ORF68 and the number of G residues within this region (nucleotides 732–739). Therefore, sequencing of ORF68 replaced the restriction fragment length polymorphism in molecular and epidemiological analyses of the EHV-1 strains obtained during outbreaks [16, 17].

The aim of this study was to analyse the neuropathogenic potential and genetic relationship of EHV-1 strains isolated from aborted equine fetuses in Poland.

## Materials and methods

### Virus strains, cell cultures, DNA extraction

**Fig. 1:**
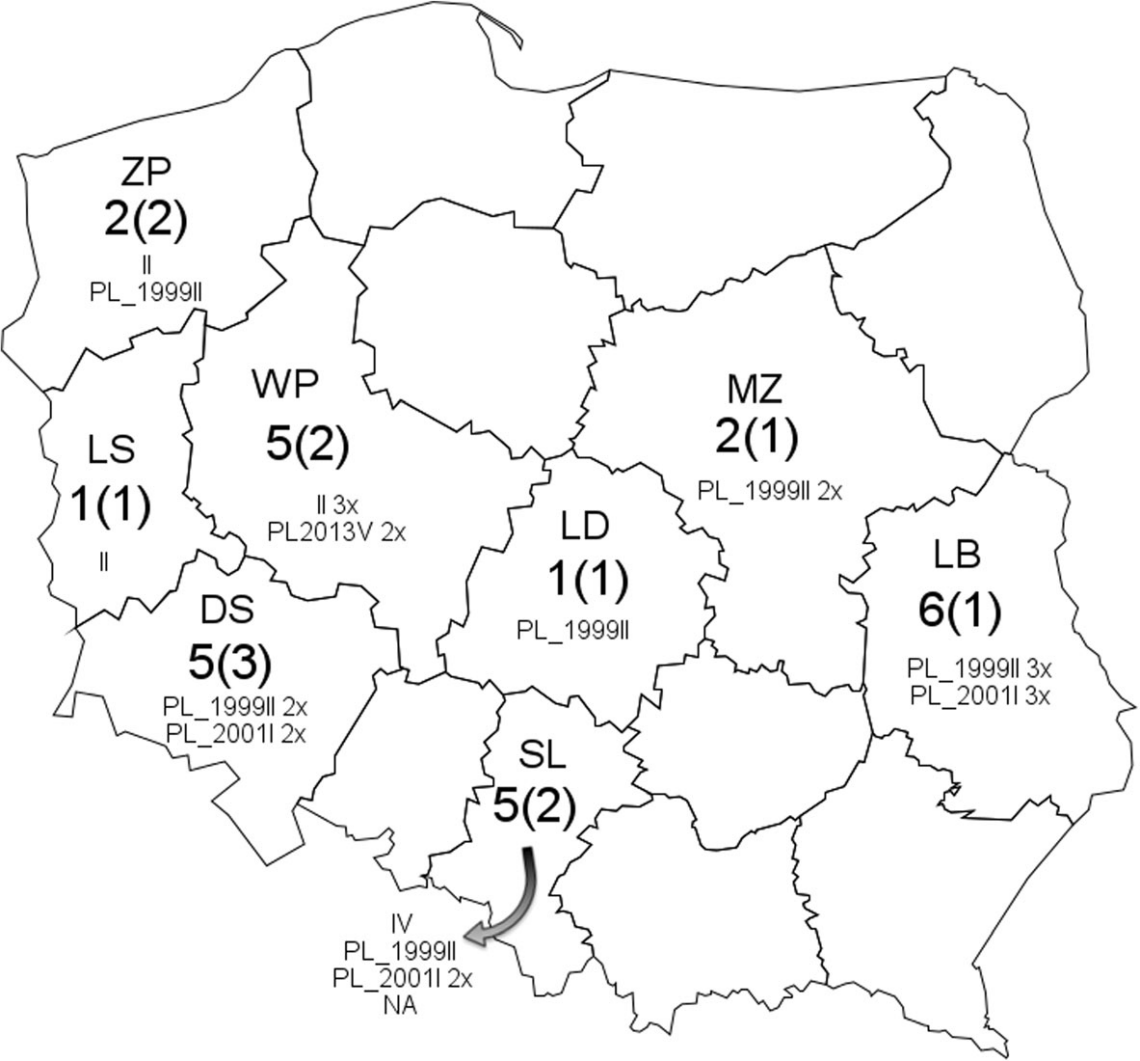
Sources of Polish EHV- 1 sequences included in the study, represented by province of origin. Total number of isolates vs. number of studs in brackets. WP- Voivodeship wielkopolskie; SL- Voivodeship slaskie; ZP- Voivodeship zachodniopomorskie; LB- Voivodeship lubelskie; DS- Voivodeship dolnoslaskie; SK- Voivodeship swietokrzyskie; LS- Voivodeship lubuskie; MZ- Voivodeship mazowieckie; LD- Voivodeship lodzkie

The 27 field strains analysed in this study were isolated from placentas or internal organs (liver, spleen, lungs) of aborted fetuses. All samples were sent as clinical samples to the Division of Microbiology, Department of Pathology, Faculty of Veterinary Medicine, Wrocław University of Environmental and Life Sciences between 1993 and 2017 (Fig. 1, Table 1). The majority of the strains originated from large or medium size breeder studs, and only a few of them from recreational studs. A vaccine strain RacH and DNA from a neuropathogenic strain Ab4 were also included in the study, as positive controls. After the original isolation, the viruses were stored as cell culture supernatants at − 80 °C or in liquid nitrogen. Before the isolation of DNA, all strains were cultured on rabbit kidney cells (RK-13) maintained in Minimum Essential Medium (MEM, Sigma-Aldrich, Germany) at 37 °C in an atmosphere containing 5% CO_2_ in T25 flasks until appearance of the cytopathic effect. The flasks were then frozen and thawed and 200 μl of supernatant was subjected to DNA extraction with the QIAmp DNA Mini Kit (Qiagen) according to the manufacturer’s instructions, with a final elution volume of 50 μl.

**Table 1:** Characteristics of isolated EHV-1 strains

Serial number of strain	Strain reference number	Date of virus isolation	Virus isolation source	Place of isolation stable/voivodeship	Group
1	93_01	16 March 1993	fetus	A/ZP	II
2	93_02	8 April 1993	fetus 10 m	A/SL	IV
3	93_03	8 April 1993	fetus 10 m	A/SL	PL_2013_V
4	93_04	8 April 1993	fetus 9 m	A/SL	partial (PL_2013_V)
5	93_05	8 April 1993	fetus 6 m	B/WP	II
6	95_01	23 January 1995	fetus	A/WP	PL_2013_V
7	95_02	28 January 1995	fetus	A/WP	II
8	96_01	12 February 1996	fetus 8 m	A/WP	PL_2013_V
9	97_01	15 January 1997	fetus 8 m	A/LB	PL_1999_II
10	97_02	23 February 1997	fetus 10 m	A/LB	PL_1999_II
11	97_03	23 February 1997	fetus 8-9 m	A/LB	PL_1999_II
12	97_04	25 February 1997	fetus 10 m	A/LB	partial (PL_2001_I)
13	97_05	25 February 1997	fetus	A/LB	partial (PL_2001_I)
14	97_06	23 March 1997	fetus 10 m	A/LB	partial (PL_2001_I)
15	99_01	20 February 1999	fetus 4 m	A/SL	partial/NA
16	99_02	22 February 1999	fetus 10 m	A/DS	partial (PL_2001_I)
17	99_03	22 February 1999	fetus 10 m	A/DS	partial (PL_2001_I)
18	00_01	10 February 2000	fetus 11 m	B/WP	II
19	00_02	27 February 2000	fetus 9 m	A/LS	II
20	01_01	28 May 2001	fetus	B/DS	PL_1999_II
21	01_02	29 October 2001	fetus 7 m	A/DS	Not tested
22	04_01	17 November 2004	fetus 8 m	B/ZP	PL_1999_II
23	05_01	21 January 2005	fetus 7 m	A/LD	PL_1999_II
24	06_01	16 February 2006	fetus 9 m	B/SL	PL_1999_II
25	10_01	23 February 2010	fetus	A/MZ	PL_1999_II
26	10_02	25 February 2010	fetus	A/MZ	PL_1999_II
27	17_01	5 March 2017	fetus	C/DS	PL_1999_II

Characteristic of isolated strains. M- months. Groups: II and IV [12], PL_1999_II and PL_2013_V [19]. Partial means that the sequence is not complete, but sequence has SNPs the same as indicated in brackets. Abbreviations of the voivodeships are explained in Fig. 1

### PCR amplification and sequence analysis

The amplification of ORF30 was performed with primers described by [13] (ORF30F: 5’-GCTACTTCTGAAAACGGAGGC-3′; ORF30R:5’-CTATCCTCAGACACGGCAACA-3′). Amplicons of 466 bp were generated with DreamTaq Green Master Mix in a 50 μl reaction volume with 200 nM of forward and reverse primer and 2 μl virus DNA template. The cycling conditions were as follows: initial denaturation at 95 °C for 3 min followed by 35 cycles of denaturation at 95 °C for 1 min, annealing at 56 °C for 1 min and extension at 72 °C for 2 min, followed by one step of final extension at 72 °C for 8 min.

The PCR products were visualized on a 2% agarose gel, bands were excised and purified with the Gel-Out kit (A&A Biotechnology, Poland), then eluted in 50 μl nuclease-free water. The purified products were digested with *Sal* I enzyme (recognition site 5′ G↓ TCGAC 3′) (EURx, Poland) in a 50 μl reaction volume containing 15 μl of purified PCR product, 5 μl 10x Buffer High, 0.5 μl 100x BSA, and 1 μl *Sal* I enzyme, for 1 h at 37 °C followed by 20 min at 65 °C. The Ab4 and RacH strains were used as positive and negative controls for *SalI* digestion, respectively. After digestion, the products were visualized by electrophoresis on 2% agarose gel. The remaining 35 μl of purified PCR products were subjected to sequencing with ORF30F primer (Genomed, Poland).

Amplification of the 645 bp region of ORF68 was performed with primers described by Nugent et al. [12] (ORF68F: 5’CAAGAAACCACTGCTCAACC3’; ORF68R: 5’AGCATTGCCAAACAGTTCC3’). The PCR was performed with Agilent’s Herculase II fusion DNA polymerase (Agilent Technologies, Santa Clara, USA), with the same conditions as described by Negussie et al. [10]. The PCR products were visualized on 2% agarose gels, the bands were excised, purified with the Gel-Out kit or by a sequencing company (A&A Biotechnology, Poland), and subjected to sequencing with at least two primer sets: ORF68F primer and for some products with ORF68R or ORFS1 primer 5’GAAGATAGAATGGGTGTGAG’3 (Genomed, Warsaw Poland; GATC Cologne, Germany). Chromatograms obtained from sequencing were manually checked for errors with FinchTV software. The fragments of ORF68 sequences obtained were aligned with a ClustalW algorithm, with a set of representative sequences of each group from the original Nugent et al. 2006 study [12] and a recent Polish study Stasiak et al. [18] obtained from GenBank with MEGA7.1 software [19]. The ORF68 sequences obtained in this study were subjected to GenBank under the accession numbers MH329902-H329927.

## Results

**Fig. 2:**
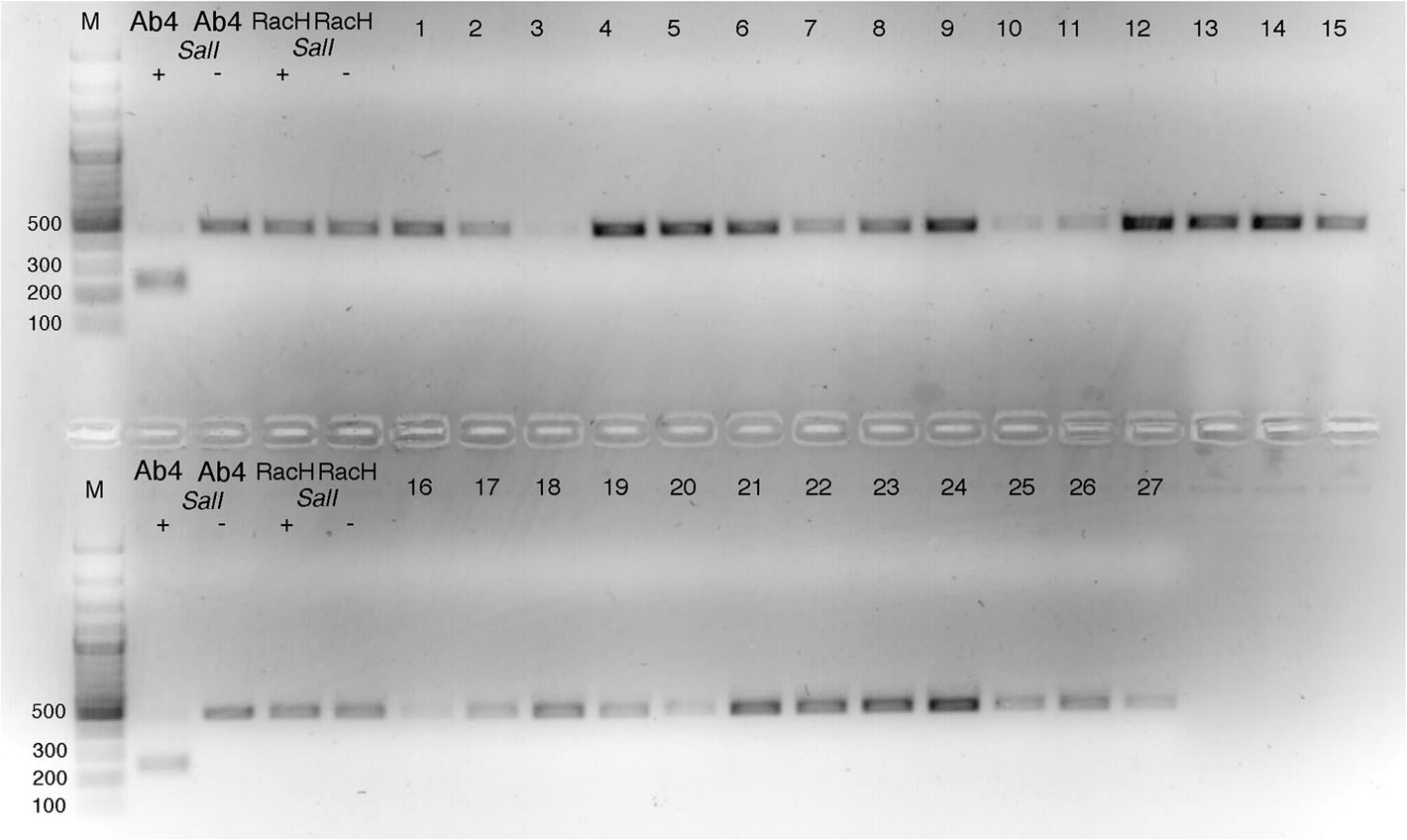
Picture of the *SalI* digestion of the PCR product of ORF30. M: molecular marker Gene Ruler Plus (Thermo), size shown in bps. 1–27 ORF30 PCR products digested with *SalI*. The numbers 1–27 correspond to the numbers of strains listed in Table 1. Ab4- neuropathogenic strain, RacH-vaccine strain, non-neuropathogenic

PCR products with a size of approximately 450 bp from 27 Polish EHV-1 strains were obtained from the ORF30 region of the virus genome. The *SalI* digestion of PCR products was negative for EHV-1 samples (Fig. 2). The sequencing of the PCR products further confirmed that all analysed sequences had adenine in position 2254. Guanidine was not observed in ORF30, meaning that all 27 strains isolated from aborted equine fetuses in Poland between 1993 and 2017 belonged to the non-neuropathogenic variant (N_752_) of EHV-1. No other mutations, including substitution C to A in position 2258, were observed in analyzed ORF30 sequences. Sequences of EHV-1 ORF30 are added in Additional file 1.

**Fig. 3:**
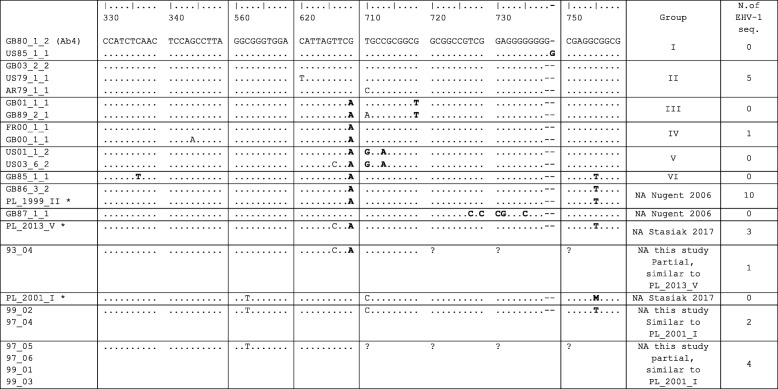
Location of single point mutations within the ORF68 gene of Polish EHV-1 sequences. Nucleotides are numbered according to accession number DQ172353.1 (GB80_1_2 Ab4 strain). Typical nucleotide positions for each groups [12] are highlighted. Asterisk: ORF68 sequences described in [18]. Dots represent the same nucleotide sequence. Dashes represent deletions. NA: not assigned

PCR products with a size of approximately 900 bp were generated from the ORF68 region of the genome including approximately 600-bp-long polymorphic segments. Products were sequenced and aligned to identify SNPs. These SNPs are presented in Fig. 3. The nucleotide sequence of the Ab4 strain (GB80_1_1) as a member of EHV-1 Group 1 served as a basis for the comparison of nucleotide changes. The sequences obtained from sample 01_02 had lower quality despite multiple sequencing efforts; therefore this strain was excluded from the analysis. The sequences obtained for 93_04, 97_05, 97_06, 99_01 and 99_03 are shorter than the others, due to poorer sequencing coverage for region nt 720–760, but were included in the analysis. Out of 26 Polish EHV-1 sequences analysed in this study, five (19.2%) belonged to group II and one (3.8%) belonged to group IV, while the remaining 20 (76.9%) were not classified within any of the groups originally described by Nugent et al. [12] (Table 1 and Fig. 3). More than half of the EHV-1 sequences (57.7%) contained A_629_ SNP. Ten Polish EHV-1 sequences (38.5%) possessed the same substitutions (A_629_ and T_750_) as the EHV-1 sequence of GB86_3_2, which was described as an unassigned sequence in the original study by Nugent et al. [12] and Polish EHV-1 sequence PL_1999_II from a recent study by Stasiak et al. [18]. Three sequences had the same SNPs (A_629_, C_626_ and T_750_) as sequence PL_2013_V, while two sequences were similar to PL_2001_I from the Stasiak et al. [18] (Figs. 2 and 3).

All of the analysed ORF68 sequences possessed seven G residues in a homopolymeric tract compared to eight G residues in the Ab4 strain (nucleotides 732–739). The number of G residues was not established for 93_04, 97_05, 97_06, 99_01 and 99_03 due to the shorter sequences available for those strains.

A clear geographical distribution is difficult to establish (Table 1) since group “PL_1999_II” sequences were found in 7 different voievodenships in contrast to the group II described by Nugent et al. [12] which was found only in western voievodenships. Also, some sequences isolated from the same stud within a similar time period differed, e.g., in the 1997 Lubelskie voievodenship (LB) outbreak isolates 97_01–97_03 contained a A_629_ SNP, vs. a T_562_ SNP of isolates 97_04 –97_06. Two EHV-1 sequences isolated in the same stud over several years (B/WP- Table 1) clustered within group II, which may indicate that a similar virus was present in that region for 7 years.

## Discussion

The strains included in this study originated from abortion cases that occurred between October and May, which is the breeding season in mares (Table 1) [4].

In this study, none of the EHV-1 strains isolated from aborted fetuses between 1993 and 2017 belonged to the neuropathogenic genotype D_752_, and all belonged to the non-neuropathogenic variant N_752_. In two recent studies conducted in Poland, the neuropathogenic variant D_752_ was found in 2 out of 20 cases (10%) and in none out of 37 (0%) EHV-1 isolates obtained from abortion cases [18, 20]. In those studies, Polish D_752_ EHV-1 strains were isolated in the years 2009 and 2010. Our study includes older isolates than previous Polish studies done by Stasiak et al. [18, 20]. Summarizing all the analyses so far, on Polish EHV-1 sequences from years 1993 to 2017, the prevalence of the neuropathogenic genotype D_752_ is very low, only 3.07% (2/65 EHV-1). In all three studies (this one and [18, 20]), the abortions were not associated with neurological symptoms in the horse premises. It seems that non-neuropathogenic EHV-1 strains are prevalent in Poland. To the author’s knowledge, EMH was never described in Poland, although horse practitioners report isolated incidents of neurological symptoms in horses, that are, however, not supported by any laboratory diagnosis of EHV-1. This is in contrast to other countries, where devastating outbreaks of EHM were reported [9, 10, 21, 22].

While studies indicate that the non-neuropathogenic N_752_ (nt A2254) variants are more common, the prevalence of D_752_ strains have increased in recent decades in the USA and some European countries [6, 15]. However, a similar increase has not been observed in other countries such as Japan or New Zealand [23, 24]. Recent studies suggest that neuropathogenic strains could have a selective advantage over non-neuropathogenic strains which have increased their prevalence in horse populations [25]. A very low prevalence of D_752_ strains and an absence of EHM outbreaks in Poland could be due to the late introduction of these strains in Poland, therefore it is recommended to monitor the genotype of EHV-1 in the future. If the D752 strains were more prevalent, this could influence quarantine rules and infectious disease management in studs. It could also influence the amount of samples sent for diagnostics.

The use of the ORF68 sequence as a molecular marker associated with the geographical origin of EHV-1 was first proposed by Nugent et al. [12]. However, more data now available for this sequence indicate that many sequences cannot be classified within already established groups. In a similar study performed in Hungary, 65.7% of EHV-1 isolates were grouped according to the classification of Nugent et al. [12] into groups II, III and IV, while the remaining isolates formed four separate groups [17]. In a recent Polish study, the main EHV-1 groups were also III and IV, while the majority of sequences were matched either by the Nugent “unassigned” group or formed a separate group [18]. In our study, analysis of the geographical distribution of strains isolated in different regions in Poland have not revealed any pattern or clustering. In fact, similar sequences have been isolated from multiple regions, and the others appear only in one stud in a particular year. These findings are in agreement with previous studies conducted in Hungary and Poland [17, 18]. In this study, we observed different EHV-1 strains that caused abortions in the same stud within a similar time period. This could suggest that at least some of these abortions might have been caused by reactivation of a persistent EHV-1 infection rather than a single introduction of a new virus into a stud’s premises (stable A/LB, Table 1). It is proposed that EMH and abortions can be caused by virus shed during the reactivation of latency and transmission to susceptible horses in the stud [3, 26]. All the recent isolates of EHV-1 sequenced in this study belong to group PL_1999_II. This particular ORF68 sequence appeared in 1997 and is the only sequence isolated since 2001, suggesting that these EHV-1 strains possess advantages in transmission or more often induce abortion in mares.

## Conclusions

In summary, equine abortion cases that occurred in Poland between 1993 and 2017 were caused by EHV-1 with a non-neuropathogenic ORF30 N_752_ genotype. On the basis of ORF68 sequences, the majority of EHV-1 strains cluster within groups II, IV or cannot be assigned to any of the known groups, but show similarity to those already described in Poland.

## Additional file

Additional file 1: Nucleotide sequences obtained from sequencing of ORF30 PCR products. Data is presented in FASTAformat. (FAS 8 kb)

## Abbreviations

EHV-1 equine herpesvirus 1: EHM- equine herpesvirus myeloencephalopathy
nt: nucleotide
ORF: Open reading frame
SNP: Single point mutation
